# Neurochemistry of the mammillary body

**DOI:** 10.1007/s00429-023-02673-4

**Published:** 2023-06-28

**Authors:** Witold Żakowski, Piotr Zawistowski

**Affiliations:** Department of Animal and Human Physiology, Faculty of Biology, University of Gdańsk, Wita Stwosza 59, 80-308 Gdańsk, Poland

**Keywords:** Mammillary body, Neuropeptides, Neurotransmitters, Rat

## Abstract

The mammillary body (MB) is a component of the extended hippocampal system and many studies have shown that its functions are vital for mnemonic processes. Together with other subcortical structures, such as the anterior thalamic nuclei and tegmental nuclei of Gudden, the MB plays a crucial role in the processing of spatial and working memory, as well as navigation in rats. The aim of this paper is to review the distribution of various substances in the MB of the rat, with a description of their possible physiological roles. The following groups of substances are reviewed: (1) classical neurotransmitters (glutamate and other excitatory transmitters, gamma-aminobutyric acid, acetylcholine, serotonin, and dopamine), (2) neuropeptides (enkephalins, substance P, cocaine- and amphetamine-regulated transcript, neurotensin, neuropeptide Y, somatostatin, orexins, and galanin), and (3) other substances (calcium-binding proteins and calcium sensor proteins). This detailed description of the chemical parcellation may facilitate a better understanding of the MB functions and its complex relations with other structures of the extended hippocampal system.

## Introduction

Many studies have been focusing on the role of the hippocampus in memory and learning processes, yet the structure does not act in isolation from other brain regions. The mammillary body (MB) is part of the extended hippocampal system, which is known for its involvement in the integration of information and mnemonic processes (Aggleton and Brown 1999). Besides the MB, the system includes also the hippocampal formation, anterior thalamic nuclei (ATN), retrosplenial, entorhinal and cingulate cortices, the tegmental nuclei of Gudden (TNG), and nerve tracts connecting the mentioned structures (Aggleton et al. 2010). Lesions of the MB in rodents have shown its critical role in memory processes (Aggleton et al. 1995; Béracochéa and Jaffard 1995; Gaffan et al. 2001; Nelson and Vann 2014; Santín et al. 1999; Sziklas et al. 1996; Vann and Aggleton 2003), whereas damage of the MB in humans is a characteristic feature of Korsakoff’s syndrome—neuropathology manifested by global amnesia (episodic/declarative memory deficits), in combination with other cognitive and behavioral dysfunction (for review see Arts et al. 2017).

The MB of rats is generally composed of two nuclei: the medial mammillary nucleus (MM) and lateral mammillary nucleus (ML), and the MM may be further divided into the medial part (MMm) and lateral part (MMl) of the medial mammillary nucleus (Allen and Hopkins 1988; Krieg 1932). The direct connections to the MB diverge from the hippocampal formation via the post-commissural fornix. However, in opposite direction, they are carried out indirectly through the anterior thalamus (Seki and Zyo 1984; Swanson and Cowan 1977). The main structure of the hippocampal formation, which innervates the MB, is the subicular complex, and these projections are unidirectional and topographically organized in rodents (Allen and Hopkins 1989; Bienkowski et al. 2018; Witter 2006). The mammillothalamic tract (mtt) is a unidirectional pathway connecting the MB with ATN and acts as the main output for the MB (Guillery 1955; Vann et al. 2007). Neurons in the MMm and MMl send their axons to the anteromedial thalamic nucleus (AM) and antero-ventral thalamic nucleus (AV), respectively; while inputs to the antero-dorsal thalamic nucleus (AD) arise from the ML (Seki and Zyo 1984; Shibata 1992; Watanabe and Kawana 1980). Another important set of the MB projections is a bidirectional connection with the TNG, conveyed via the mammillotegmental tract, which is composed of descending collaterals of MB axons running in the mtt, and the mammillary peduncle—the ascending branch. The organization of these projections is well established: the MM is connected with the ventral tegmental nucleus (TNGv), and the ML with the dorsal tegmental nucleus (TNGd) (Allen and Hopkins 1989; Hayakawa and Zyo 1989; 1991).

Each part of the MB plays a specific role in the extended hippocampal system. For example, studies on rats have shown that the MM, especially its lateral part, exerts modulatory effects on hippocampal theta rhythm (via the projections to the AV), while the ML constitutes part of the head direction (HD) system (together with the AD) and therefore participates in navigation (for review see Aggleton et al. 2010). With all things considered, the MB serves a critical function in memory and spatial navigation, as a part of the system of structures interconnected to the hippocampus.

Despite obvious differences in cell morphology among various parts of the MB, the view that there is only one type of neuron within the MB is well established (e.g., Allen and Hopkins 1988; Powell and Cowan 1954; Veazey et al. 1982; Takeuchi et al. 1985). However, many years of histochemical investigations have shown that the heterogeneity of MB neurons is much more extensive, and a recent study by Mickelsen and colleagues (2020) seems to support this notion at a molecular level as well. The following article is the first attempt to review and summarize the neurochemical parcellation of the mammillary body by describing the distribution of various substances in this structure of the rat, with a commentary on other species’ brains where necessary. The article is divided into three sections: the first one is devoted to the classical neurotransmitters, the second section focuses on various neuropeptides, and the third one reviews the presence of calcium-related proteins. Two tables which summarize the chemical organization of the rat MB are included in the paper: Table 1 describes the intrinsic neurochemistry of MB neurons, while Table 2—the neurochemistry of innervations of the MB.

**Table 1:** The intrinsic neurochemistry of the mammillary body in rats—relative quantity of classical neurotransmitters (or their markers), neuropeptides, calcium-binding proteins, and calcium sensor proteins in the neurons

	MM		ML	Additional remarks	References
	MMm	MMl			
Neurotransmitters					
Glutamate	+ + +			~ 60% of all neurons projecting to the ATN; likely source of glutamate in the TNG	Gonzalo-Ruiz et al. (1996; 1998)
NAAG	+ + +	+ +	+	Abundant in the whole extended hippocampal system	Tsai et al. (1993)
GABA (and GAD)	–			–	Benson et al. (1992); Gonzalo-Ruiz et al. (1993); Sakaue et al. (1988)
Acetylcholine (ChAT)	–			–	Tago et al. (1987); Ruggiero et al. (1990)
Serotonin	–			–	Moore et al. (1978); Steinbusch and Nieuwenhuys (1981)
Dopamine (TH)	–			–	Chan-Palay et al. (1984); Gonzalo-Ruiz et al. (1992a)
Neuropeptides					
Leu-enkephalin	+ + +	+	+ +	~ 50% of all neurons projecting to the ATN; source of leu-enkephalin in the TNG	Finley et al. (1981); Fujii et al. (1987); Gonzalo-Ruiz et al. (1998); Khachaturian et al. (1983); Lantos et al. (1995); Yamano and Tohyama (1987)
Substance P	–			–	Larsen (1992); Shults et al. (1984); Warden and Young (1988)
CART	+ + +		+ +	Likely source of CART in the ATN	Douglass et al. (1995)*; Koylu et al. (1997); Hurd and Fagergren (2000)*
Neurotensin	–			–	Kahn et al. (1982)
Neuropeptide Y	–			–	de Quidt and Emson (1986); Ni et al. (2015)
Somatostatin	–			–	Johansson et al. (1984); Lantos et al. (1995)
Orexins	–			–	Cutler et al. (1999); Nixon and Smale (2007)
Galanin	–			–	Lantos et al. (1995); Skofitsch and Jacobowitz (1985); Takatsu et al. (2001)
CaBPs					
Calretinin	–		+ + +	Source of calretinin in the ATN	Jacobowitz and Winsky (1991); Resibois and Rogers (1992); Rogers and Resibois (1992)
Calbindin	+ + +		–	Likely source of calbindin in the ATN	Celio (1990); Rogers and Resibois (1992); Sequier et al. (1990)
Parvalbumin	+ +		+	–	Celio (1990)
NCSPs					
NVP-3 and hippocalcin	+ + +		+	One of the richest presence in the hypothalamus	Paterlini et al. (2000)*; Takami et al. (1985);
Frequenin, NVP-1 and NVP-2	+ / + +			–	Paterlini et al. (2000)*; Saitoh et al. (1994)

*ATN* anterior thalamic nuclei, *CaBPs* calcium-binding proteins, *CART* cocaine- and amphetamine-regulated transcript, *ChAT* choline acetyltransferase, *GABA* gamma-aminobutyric acid, *GAD* glutamic acid decarboxylase, *ML* lateral mammillary nucleus, *MM* medial mammillary nucleus, *MMm* medial part of the MM, *MMl* lateral part of the MM, *NAAG* N-acetyl-aspartyl-glutamate, *NCSPs* calcium sensor proteins, *TH* tyrosine hydroxylase, *TNG* tegmental nuclei of Gudden

*mRNA distribution study

Protein or mRNA presence: +  +  + high; +  + moderate; + low; – absent

**Table 2:** The neurochemistry of innervations of the mammillary body in rats—relative quantity of classical neurotransmitters (or their markers), neuropeptides, calcium-binding proteins, and calcium sensor proteins in the neuropil, i.e., fibers and axon terminals

	MM		ML	Additional remarks	References
	MMm	MMl			
Neurotransmitters					
Glutamate	+ + +			Source: the subiculum	Gonzalo-Ruiz et al. (1996); Umaba et al. (2021)
VGluT1	+ + / + + +		+ / + +	–	Fremeau et al. (2001); Kaneko et al. (2002); Sakata-Haga et al. (2001)
VGluT2	+		+ + +	–	
NAAG	+ + +	+ +	+	Abundant in the whole extended hippocampal system	Tsai et al. (1993)
GABA	+ +	+ + +	+ + +	Source: the TNG	Gonzalo-Ruiz et al. (1993; 1996; 1999); Sakaue et al. (1988); Wirtshafter and Stratford (1993)
Acetylcholine (ChAT)	+ +	+	+ +	Source: the laterodorsal tegmental nucleus	Gonzalo-Ruiz et al. (1999); Ruggiero et al. (1990)
Serotonin	+ +	+	+	Source: the median and dorsal raphe nuclei	Azmitia and Segal (1978); Moore et al. (1978); Steinbusch and Nieuwenhuys (1981)
Dopamine (TH)	+ +	+	+ + +	Source: the supramammillary nucleus	Chan-Palay et al. (1984); Gonzalo-Ruiz et al. (1992a)
Neuropeptides					
Leu-enkephalin	+ +		+	Source: the TNG	Finley et al. (1981); Gonzalo-Ruiz et al. (1998); Khachaturian et al. (1983); Lantos et al. (1995)
Substance P	−/ +			Source: the TNG	Gonzalo-Ruiz et al. (1999); Lantos et al. (1995); Larsen (1992); Shults et al. (1984)
CART	+				Koylu et al. (1997)
Neurotensin	+			Much higher in young rats; source: the subiculum	Kahn et al. (1982); Kiyama et al. (1986)
Neuropeptide Y	+			–	de Quidt and Emson (1986); Ni et al. (2015)
Somatostatin	+			–	Johansson et al. (1984); Lantos et al. (1995)
Orexins	+		++	–	Cutler et al. (1999); Nixon and Smale (2007)
Galanin	−/ +			–	Lantos et al. (1995); Skofitsch and Jacobowitz (1985); Takatsu et al. (2001)
CaBPs					
Calretinin	+		+ + +	Source: the postsubiculum	Celio (1990); Jacobowitz and Winsky (1991); Resibois and Rogers (1992); Rogers and Resibois (1992); Dillingham et al. (2015)
Calbindin	+ + +		–	Source: the TNG	
Parvalbumin	+ + +		+		
NCSPs					
NVP-3 or NVP-1	–	+ + +	–	Referred to as visinin	Takami et al. (1985)
Frequenin, hippocalcin and NVP-2	n/a			–	

*CaBPs* calcium-binding proteins, *CART* cocaine- and amphetamine-regulated transcript, *ChAT* choline acetyltransferase, *GABA* gamma-aminobutyric acid, *ML* lateral mammillary nucleus, *MM* medial mammillary nucleus, *MMm* medial part of the MM, *MMl* lateral part of the MM, *NAAG* N-acetyl-aspartyl-glutamate, *NCSPs* calcium sensor proteins, *TH* tyrosine hydroxylase, *TNG* tegmental nuclei of Gudden, *VGluT* vesicular glutamate transporters

Protein presence: +  +  + high; +  + moderate; + low; – absent; n/a no data

## Classical neurotransmitters

In this section, the presence of the classical neurotransmitters in the MB will be described. To date, various neurotransmitters (or their markers), along with their receptors, have been found in the MB of the rat, i.e., glutamate (and other excitatory transmitters), gamma-aminobutyric acid, acetylcholine, serotonin, and dopamine.

### Excitatory neurotransmitters

Glutamate (Glu) is the principal excitatory neurotransmitter in the central nervous system (CNS) and aspartate (Asp) is a selective agonist for glutamate receptors (Kubrusly et al. 1998). Some studies have shown that Asp has characteristics of a classical neurotransmitter (Cavallero et al. 2009; Gundersen et al. 1998; but see Herring et al. 2015), thus its distribution will be described in the following section. Excitatory amino acids, such as Glu and Asp, are implicated in several crucial functions of the nervous system, including memory (for review see McEntee and Crook 1993; Reis et al. 2009; Riedel et al. 2003). Glutamate receptors consist of both ionotropic: α-amino-3-hydroxy-5-methyl-4-aspartate receptors (NMDA), α-amino-3-hydroxy-5-methyl-ioxyzole-4-propionic acid receptors (AMPA), kainate receptors (KA), and metabotropic receptors (Hollmann and Heinemann 1994; Mayer and Westbrook 1987; Salt and Eaton 1996).

Glu and Asp have been found in a substantial number of cell bodies in the rat MB, where both substances co-localized (Gonzalo-Ruiz et al. 1996). Moreover, gene expression analysis has shown that all parts of the murine MB contain neurons expressing genes required for glutamate synthesis and packaging, indicating their excitatory function (Mickelsen et al. 2020). Almost all MB neurons send their axons to the ATN (Aggleton et al. 2010; Vann et al. 2007), and 50–60% or even more of these neurons have been shown to contain Glu/Asp (Gonzalo-Ruiz et al. 1998). Indeed, in all subnuclei of the anterior thalamus, Glu/Asp positive fibers and terminal-like boutons have been found (Gonzalo-Ruiz et al. 1996). It seems that the signal processing between these two structures (as well as along the extended hippocampal system) is highly dependent on Glu/Asp transmission. The same neurons of the MB may also be a source of excitatory inputs for the TNG, as descending axons in the mammillotegmental tract are collaterals of ascending axons in the mtt (Hayakawa and Zyo 1989). It has been shown that most of the mammillary axon terminals in both, the TNGv and TNGd, contain round vesicles and make asymmetric synaptic contacts, which is the characteristic feature of excitatory synapses. These terminals are small (diameter < 2 μm) with synapses located mostly on small-diameter dendrites (Allen and Hopkins 1990), which is an interesting difference from the terminals of the mammillothalamic collaterals—large in size (diameter 1.5–4.0 μm) and forming synapses primarily on proximal and secondary dendrites (Dekker and Kuypers 1976).

All MB subnuclei also contain many Glu/Asp-positive fibers and terminal-like structures (Gonzalo-Ruiz et al. 1996). There is strong evidence that the subiculum is the main source of this Glu/Asp input. First, axonal terminals from the subiculum contain round vesicles and form asymmetric synaptic junctions on mammillary neurons, thus are excitatory (Allen and Hopkins 1989). Second, a surgical lesion of the fornix heavily reduced high-affinity uptake of Glu/Asp in the rat MB, but did not affect other substances’ uptake, such as gamma-aminobutyric acid, glutamic acid decarboxylase, or choline acetyltransferase (Storm-Mathisen and Woxen Opsahl 1978; Walaas and Fonnum 1980). Recently, Umaba et al. (2021) have shown that subicular projections terminate on Glu-containing neurons of the MM, which project to the AV/AM and TNGv. The authors concluded, that this subiculum–MB connection may support information flow through the hippocampal–mammillothalamic and hippocampal–mammillotegmental circuits, using Glu as the main transmitter. Gonzalo-Ruiz et al. (1999) have revealed that a very small population of neurons projecting from the TNG to the MB contains Glu as well, but this scant level likely correlates with a metabolic role of Glu rather than with a neurotransmission role.

Interestingly, a high level of *N*-acetyl-aspartyl-glutamate (NAAG) has also been found in cell bodies and neuropil of the MB, especially in the MM–only faintly immunostained cells were found in the ML (Tsai et al. 1993). Dipeptide *N*-acetyl-aspartyl-glutamate is the second most widely distributed excitatory transmitter in the brain, which activates a limited subpopulation of NMDA receptors, as well as mGluR3. It has been proposed that NAAG may be released under conditions of elevated activation of neuronal circuits (Neale and Yamamoto 2020). It seems that NAAG has an important role in learning and memory processes as it has been found in the other structures of the extended hippocampal system, such as the hippocampal formation, anterior thalamus, retrosplenial cortex, and the main tracts of the system, i.e., the fornix, mtt and cingulum (Moffett and Namboodiri 1995; Tsai et al. 1993).

The abundant presence of vesicular glutamate transporters (VGluTs) within the MB also strongly suggests its excitatory nature. These transporters are restricted to nerve endings and are thought to regulate the amount of Glu released into the synaptic cleft (Wilson et al. 2005). Interestingly, there is a high variation of the expression arrangement across the CNS within the three isoforms of VGluTs, i.e., VGluT1-VGluT3 (Fremeau et al. 2001; Herzog et al. 2004). In the rat MB, expression of VGluT1 and VGluT2 was detected in a kind of complementary fashion. However, various studies have brought slightly different results. On the whole, immunohistochemical studies revealed that VGluT1 was present at a moderate to high level in the MM and a low to moderate level in the ML, whereas VGluT2 was much more abundant in the ML, with only small amounts in the MM (Fremeau et al. 2001; Kaneko et al. 2002; Sakata-Haga et al. 2001). Regarding VGluTs mRNA, a low expression level of VGluT1 has been found in the ML, whereas VGluT2 mRNA has shown a moderate to high expression level in the ML, and a much lower one in the MM (Hisano et al. 2000; Lin et al. 2003; Ziegler et al. 2002). A study on mice has revealed that this population of VGluT2 neurons in the ML receives direct inputs from GABAergic neurons of the TNGd, which express a high level of 5-HT1AR–serotonergic receptors involved in the regulation of wakefulness. In this model, a population of ML glutamatergic neurons would be a center that promotes wakefulness, through the network involving inhibition of GABAergic neurons in the TNGv by serotonergic neurons of the median raphe nucleus (via 5-HT1AR), and, as result, disinhibition of ML neurons (Chazalon et al. 2018).

It has been proposed that VGluT1 and VGluT2 reflect different subclasses of Glu terminals, which might differ in some aspects of the packaging and regulating the release of Glu (Fremeau et al. 2001; Pietrancosta et al. 2020). For example, excitatory projections from the subiculum to the MB, which collateralize to innervate the retrosplenial cortex, overlap with VGluT2, but not VGluT1 presence (Kinnavane et al. 2018). The exact nature of the differences between these two VGluT isomers is still debated, but if they co-expressed in the same vesicles, then that would lead to the increased volume of Glu inside a single vesicle in comparison to a vesicle equipped only with a singular isoform of VGluT (Schuske and Jorgensen 2004). If such co-localization was present in the MB, it would likely involve the ML. First, the ML contains higher amounts of both VGluT isomers, with respect to protein, as well as mRNA. Second, such co-localization is present in a huge population of perikarya and many punctate structures in the AD in rats (Barroso-Chinea et al. 2007; Oda et al. 2014), which is the main target of ML projections. Possibly, the presence of two isoforms may be related to a specific function of the nucleus, i.e., contribution to the HD system. Both nuclei, the ML and AD, contain a substantial percent of HD cells that fire as a result of the specific pattern of the animal’s head movement in a horizontal plane (for review see Taube 2007). The other target of MB projections, i.e., TNG, contains moderate amounts of VGluT2, but virtually none of VGluT1 (Kaneko et al. 2002).

Both ionotropic and metabotropic glutamate receptors are present in the rat MB. Van den Pol (1994) have found that most neurons of the MB express the NR1 subunit of NMDA receptors; however, it was GluR1 and GluR2 which showed the highest expression level among ionotropic receptors in the MB (Gold et al. 1997; Gu et al. 2008; Martin et al. 1993; Ohishi et al. 1998; Sato et al. 1993; Van den Pol 1994). GluR1 is also the most highly expressed AMPA subunit receptor in the ATN (Gold et al. 1997; Martin et al. 1993; Sato et al. 1993; Spreafico et al. 1994). There are some discrepancies among various studies, but generally, the expression of GluR3 and GluR4 mRNA is very low within the rat MB (Gold et al. 1997; Martin et al. 1993; Sato et al. 1993; Van den Pol et al. 1994). Across kainate receptor subunits, GluR6 mRNA showed a moderate to high expression, but there was virtually a lack of GluR5 and GluR7 mRNA in the MB (Van den Pol et al. 1994).

One study has shown that the human MB contains a high level of Glu/Asp (Banay-Schwartz et al. 1992), which is also true for some other structures of the hippocampal system, such as the hippocampus and ATN (Banay-Schwartz et al. 1992; Popken et al. 2002).

### Gamma-aminobutyric acid

Gamma-aminobutyric acid (GABA) is the main inhibitory neurotransmitter in the mammalian brain. Two types of GABA receptors have been described: ionotropic GABA_A_ receptors and slower, metabotropic GABA_B_ receptors (see Bowery et al. 1987).

According to morphological studies, there is little evidence for interneurons in the MB of the rat (Allen and Hopkins 1988; Seki and Zyo 1984; Takeuchi et al. 1985). Similarly, immunohistochemical studies on rats have brought no evidence that MB neurons contain GABA. However, GABA-positive staining has been associated with axons traversing the MB and their terminals, especially in the ML and MMl (Gonzalo-Ruiz et al. 1993, 1996; Sakaue et al. 1988). It has been additionally confirmed in the study conducted by Benson et al. (1992), in which no glutamic acid decarboxylase (GAD; an enzyme synthesizing GABA) RNA was detected in the MB, while gene expression mapping has failed to find any GABAergic neuronal marker in the murine MB (Mickelsen et al. 2020). With regard to GABAergic receptors, a moderate to high expression level of various subunits of GABA_A_ receptor has been found within the rat MB (Fritschy and Mohler 1995; Pirker et al. 2000). However, GABA_B_ receptor RNA showed only weak expression, confined to the MM (Durkin et al. 1999).

Most likely, the GABAergic components observed in the MB have their source in the TNG. First, axon terminals from the tegmental nuclei are characterized by pleomorphic vesicles and form symmetric synaptic junctions on the majority of MB neurons, presumably exerting inhibitory effects (Allen and Hopkins 1989; Hayakawa and Zyo 1991). Second, Gonzalo-Ruiz et al. (1999) have shown that neurons of the TNGv and TNGd are GABA-positive, whereas lesions of these two tegmental nuclei in rats resulted in a substantial decrease in the number of GAD-positive fibers and terminals within the MM and ML, respectively (Wirtshafter and Stratford 1993). The GABAergic inputs from the tegmental nuclei innervate mostly excitatory neurons in the MB of rodents and may be seen as an inhibitory feedback loop which controls functions of the MB (Wirtshafter and Stratford 1993) and, in consequence, other structures of the extended hippocampal system. Lesions of the TNGv in rats impaired the performance of several memory tasks, such as delayed-matching-to-place in the water maze, the T-maze alternation task, and working memory in the radial arm maze—all of which are also sensitive to the damage of the MB, mtt, ATN and the hippocampus (Vann 2009). Similar to MM neurons, neuronal cells in the TNGv fire rhythmically in a fashion corresponding to hippocampal theta rhythm, and it has been proposed that the inhibitory influence of the midbrain inputs may moderate theta activity in the MB (Kocsis et al. 2001; Vertes et al. 2004). In such case, any damage to the TNGv may produce a desynchronization of activity (i.e., a lack of functional coupling) of various structures within the extended hippocampal system, which, as result, may lead to memory deficits in rodents, as well as in humans (Goldberg et al. 1981). Likewise, lesions of the TNGd produce impairments of the spatial task performance in rats, but in this regard, a disruption in the acquisition of navigational strategies is involved (Clark et al. 2013). The TNGd is a part of the HD system, and damage to this nucleus completely suppresses the HD signal in the AD—a structure that is only indirectly connected with the TNGd, via the ML (Bassett et al. 2007). Thus, the direct inhibitory projections from the TNGd to the ML seem to be necessary for an animal to navigate toward specific places in an environment.

The lack of a developed local inhibitory network is a common feature for the MB and anterior thalamus, as no interneurons have been found in the ATN in rats (Benson et al. 1992; Wang et al. 1999). While the MB receives its GABAergic inputs from the tegmental nuclei, the activity of the ATN is inhibited by the projections from the thalamic reticular nucleus (Gonzalo-Ruiz and Lieberman 1995). It was proposed that the number of GABAergic interneurons in the mammalian thalamus is related to behavior complexity. The more complex the behavior of a species, the higher the number of GABAergic interneurons (Arcelli et al. 1997). Indeed, an increase in the number of GABAergic neurons has been observed in the ATN of cats, non-human primates, and humans (for review see Żakowski 2017). In contrast, there is a lack of interneurons in the MB of the cat and rabbit (Guillery 1955), but a small population of inhibitory neurons has been detected in the MB of the rhesus monkey (Xiao and Barbas 2002) and human (Bernstein et al. 2007; Dixon et al. 2004; Mackay et al. 1978), approximately 2% of the total neuron population in the latter.

### Acetylcholine

Acetylcholine plays an essential role in various brain functions, such as arousal, sleep, learning and memory, and many others (Woolf 1991; Woolf and Butcher 1986). Acetylcholine receptors consist of two major types: the metabotropic muscarinic receptor and the ionotropic nicotinic receptor (for review see Eglen 2006; Albuquerque et al. 2009). Two enzymes related to acetylcholine, i.e., choline acetyltransferase (ChAT) and acetylcholinesterase (AChE), are the most specific markers of cholinergic neurons, commonly used in the studies of cholinergic neurotransmission in the CNS.

While no cell bodies containing ChAT have been found in the MB of the rat, many fibers and terminal-like varicosities were concentrated primarily in the ML, but also in the MMm (Tago et al. 1987; Ruggiero et al. 1990). The most likely origin of these ChAT-positive structures is the laterodorsal tegmental nucleus, which sends its cholinergic projections toward the MB, especially the ML (Gonzalo-Ruiz et al. 1999). Terminals from the laterodorsal tegmental nucleus are characterized by pleomorphic vesicles and make symmetric synaptic connections with ML neurons, presumably constituting an inhibitory input (Hayakawa and Zyo 1992). The laterodorsal tegmental nucleus is well known for the vast presence of cholinergic neurons (Honda and Semba 1995), and is also the main source of cholinergic innervation in the anterior thalamus (Gonzalo-Ruiz et al. 1995; Hallanger et al. 1987; Holmstrand and Sesack 2011). Interestingly, a significant number of muscarinic receptors in the ATN are presynaptic, and it has been suggested that cholinergic innervation of the ATN may provide a classical presynaptic inhibition through these receptors during the activation of projections from the MB (Sikes and Vogt 1987). Regarding acetylcholine receptors in the MB, the highest concentrations of nicotinic receptors have been observed in the ML (Block and Billiar 1981; Clarke et al. 1985; Härfstrand et al. 1988; Tribollet et al. 2004).

The lack of neurons containing cholinergic markers is also a characteristic feature of other mammals’ MB, such as cats, macaques, and humans (Tago et al. 1987; Woolf 1991). Only one study has shown a low to moderate concentration of ChAT and AChE in the human MB (Mackay et al. 1978).

### Serotonin

Serotonin (5-hydroxytryptamine, 5-HT) is a crucial transmitter for various emotional, motor, and cognitive functions. Seven families of 5-HT receptors (5-HT_1-7_) have been described (for review see Barnes and Sharp 1999).

The presence of 5-HT in the rat MB is related to neuropil only, and its source could be tracked to the population of serotonergic neurons in the midbrain raphe nuclei. Moderately dense 5-HT innervation has been found in the MMm, and a significantly lower one in the MMl and ML (Moore et al. 1978; Steinbusch and Nieuwenhuys 1981). Both the median and dorsal raphe nuclei project to the MB through two pathways: the raphe medial tract reaches the MMm, while the tract overlapping with the mammillotegmental tract runs toward MMl (Azmitia and Segal 1978). A study on mice has revealed that the serotonergic projections from the median raphe nucleus may influence the activity of the MB (the ML in particular) also indirectly, via the TNGv (Chazalon et al. 2018), which was described earlier in this paper. In regard to the anterior thalamus of rat, the AV appears to be the strongest 5-HT-positive nucleus (for review see Żakowski 2017), however in other mammals, for example, the cat, this characteristic is associated primarily with the AD (Leger et al. 2001).

Serotonin receptors exhibit several distinct subtypes across the rat MB. Among them, 5-HT_7_ subtype showed the highest concentration, and it was present almost exclusively in the MM (Gustafson et al. 1996; Heidmann et al. 1998; Kinsey et al. 2001; Martin-Cora and Pazos 2004). 5-HT_7_ is known for its effects exerted on various processes related to cognition, such as learning and memory (for review see Meneses 2014; Stiedl et al. 2015). This subtype of the serotonin receptor is also the most abundant within the rat ATN (Gustafson et al. 1996; Kinsey et al. 2001; Neumaier et al. 2001). In vivo studies on the thalamic slices have shown that these receptors mediate serotonin influence on the excitability of AD neurons (Chapin and Andrade 2001). Among other subtypes, various 5-HT_1_ receptors (5-HT_1A_, 5-HT_1C_, 5-HT_1D_), as well as 5-HT_2_, showed a low to moderate level of expression in the entire rat MB (Abramowski et al. 1995; Bruinvels et al. 1993; Pazos et al. 1985; Pazos and Palacios 1985; Pompeiano et al. 1992; Wright et al. 1995).

### Dopamine

Dopamine (3,4-dihydroxyphenethylamine, DA) transmission is linked to many vital functions, such as learning, motivation, planning, and general motor control (Chinta and Andersen 2005; Lerner et al. 2021). The most distinct population of DA neurons is located in the ventral mesencephalon (around 90% of all dopaminergic cells in the brain), which gives rise to several DA subsystems, i.e., the nigrostriatal, mesolimbic, and meso-cortical pathways (Chinta and Andersen 2005). DA is synthesized with the involvement of tyrosine hydroxylase (TH), which is a commonly used marker of dopaminergic neurons in the hypothalamus and beyond. The receptors for the DA can be divided into two distinct families based on properties and structure: the D1 family (consisting of D1 and D5) and the D2 family (consisting of D2, D3, and D4). Interestingly, D2 and D3 are located in the post- and presynaptic cells, whereas D1 and D5 only in the postsynaptic ones (for review see Klein et al. 2019).

No cell bodies containing TH have been found in the MB of the rat. However, TH-positive fibers and terminals are present, but unevenly distributed within the structure. The ML is characterized by the highest number of varicose fibers and terminals, while a moderate density of axons and terminals is present in the MM, mostly in the dorsal part of the MMm and MMl (Chan-Palay et al. 1984; Gonzalo-Ruiz et al. 1992a). Most likely, the principal source of this dopaminergic innervation is the supramammillary nucleus, as it has been shown that numerous TH-positive neurons of this nucleus send dense projections to both nuclei of the MB, in particular to the ML (Gonzalo-Ruiz et al. 1992a; b). The supramammillary nucleus projects to various structures of the extended hippocampal system, including the anterior thalamus and hippocampus, as well as the septum and diagonal band of Broca, and it is pivotal to the generation of the hippocampal theta rhythm (Pan and McNaughton 2004). Interestingly, it has been suggested that the dopaminergic input to the lateral septum and MB may come from the same supramammillary nucleus neurons whose axons bifurcate on the way (Gonzalo-Ruiz et al. 1992a).

The ML is also the nucleus with the highest expression of dopamine receptors within the rat MB, especially in regard to D2 receptor (Bouthenet et al. 1987; Tiberi et al. 1991; Weiner et al. 1991). There is virtually a lack of D1 receptors in the MB (Dawson et al. 1986; Savasta et al. 1986; Weiner et al. 1991), but Tiberi et al. (1991) have found overlapping expression of D1B subtype and D2 receptor mRNA in the ML. Gurevich and Joyce (1999) have also revealed high expression of D3 receptor in the whole MB–ATN axis, i.e., the MB, mtt, and anterior thalamus. Interestingly, D2-binding sites were not present in the mtt, which suggests that D3 receptors may play important role in regulating the activity of the axis. The importance of D3 receptors seems to be conserved across species, as a similar organization of dopamine receptors was observed in the human brain, i.e., a high concentration of D3 binding sites in the MB, mtt, and anterior thalamus (Gurevich and Joyce 1999). While both the structures also contain D3 receptor mRNA in humans, other dopamine receptors showed somewhat lower expression in the MB–ATN axis (Camps et al. 1989; Gurevich and Joyce 1999). Regarding D1 receptors, however, the human MB is significantly richer in binding sites than the MB of the rat (Cortés et al. 1989). Similar to the rat, there is a lack of cell bodies containing TH in the MB of humans (Sanghera et al. 1995), however, a moderate level of TH activity has been detected (Macay et al. 1978).

## Neuropeptides

Many studies have revealed a presence (or lack of it) of various neuropeptides in the MB of the rat, such as enkephalins, substance P, cocaine- and amphetamine-regulated transcript, neurotensin, neuropeptide Y, somatostatin, orexin, galanin, and their distribution will be described in the following section.

### Enkephalins

Enkephalins (ENKs) are a group of endogenous opioids. Several ENKs have been described so far, but only leucine enkephalin (leu-ENK) has been detected in the MB of the rat (Khachaturian et al. 1985).

In the rat MB, many scattered neurons containing leu-ENK have been found in the MMm, but also in the ML (Finley et al. 1981; Fujii et al. 1987; Gonzalo-Ruiz et al. 1998; Khachaturian et al. 1983; Lantos et al. 1995; Yamano and Tohyama 1987). These cells are the source of leu-ENK in the main targets of the MB projections, i.e., the ATN and TNG, as ENK-positive fibers have been seen to enter the mtt and continue throughout its entire course to the AD and AV, where the dense accumulation of fibers and terminal boutons were present (Fujii et al. 1987; Khachaturian et al. 1983). Gonzalo-Ruiz et al. (1998) have shown that leu-ENK is present in 40–50% of neurons in the ML and MM with projections directed to the ATN, whereas lesions of the MM caused the reduction of ENK-positive fibers in the AV (Fujii et al. 1987). Moreover, the collaterals of axons projecting to the ATN which build up the mammillotegmental tract, provide an ENK input to the TNG, especially the TNGv (Yamano and Tohyama 1987). On the other hand, ENK-positive fibers and terminals are also present in the MB, but are mainly confined to the MM (Finley et al. 1981; Khachaturian et al. 1983; Lantos et al. 1995). Interestingly, Gonzalo-Ruiz et al. (1999) have demonstrated that these leu-ENK-positive structures, or at least part of them, have their origins in both the TNGv and TNGd, where many neurons containing ENK have been detected (Finley et al. 1981; Khachaturian et al. 1983). The abundance of leu-ENK in the MB–ATN axis and beyond demonstrates the high importance of this particular endogenous opioid in an information flow within the extended hippocampal system in rats. Some studies indicate that leu-ENK may play important role in the modulation of inhibitory transmission, especially in the hippocampus (for review see Drake et al. 2007).

However, the distribution of leu-ENK in the MB–ATN axis of rats is significantly different from the one observed in humans: there is a lack of enkephalins and their precursors, except for a low to moderate density of ENK-positive fibers in both, the MB and ATN (Alelú-Paz and Giménez-Amaya 2007; Bouras et al. 1984; Dudás and Merchenthaler 2003; Hurd 1996; Pittius et al. 1984; Sánchez et al. 2016; Sukhov et al. 1995).

### Substance P

One of the most well-known member of the tachykinin family–substance P (SP) is commonly found in the mammalian CNS. This neuropeptide takes a part in the regulation of various physiological and cognitive functions, including memory and learning processes (for review see Severini et al. 2002).

No cell bodies, and only a very small number of fibers and terminal boutons containing SP, have been observed in the MB–ATN axis of the rat (Lantos et al. 1995; Larsen 1992; Shults et al. 1984; Warden and Young 1988). One study suggested that SP-positive structures detected in the ML may come from neurons of the TNGd (Gonzalo-Ruiz et al. 1999). Regarding SP receptors, a moderate density has been found in the MMl and AD, and lower one in the AV and AM (Buck et al. 1986), however, Maeno et al. (1993) have failed to detect any mRNA of NK1R (SP selective receptor) within the MB–ATN axis.

Taking into account very low concentrations of SP also in the human MB and ATN (Cooper et al. 1981; Dudas and Merchenthaler 2006; Ghatei et al. 1984; Langevin and Emson 1982), it seems that this neuropeptide does not play an important role in the MB–ATN axis.

### Cocaine- and amphetamine-regulated transcript

Cocaine- and amphetamine-regulated transcript (CART) is thought to participate in many neurophysiological functions, from food intake to stress response (for review see Rogge et al. 2008). Several studies on rodents have shown that CART peptide may also be involved in memory-related behavior (e.g., Upadhya et al. 2011; Yermolaieva et al. 2001).

There is a variable density of CART-positive cells across the MB of rats. A high density of perikarya containing CART peptide is located in the MM and a moderate one in the ML, together with a low density of CART-positive fibers within the whole MB (Koylu et al. 1997). Żakowski et al. (2014) have revealed that CART-positive neurons are also present in the MB of the guinea pig, but only in the MMl, whose axons run toward the anterior thalamus. In the ATN of both, the guinea pig and rat, a high density of terminal-like structures immunoreactive for CART peptide has been detected, mostly in the AV (Koylu et al. 1998; Żakowski et al. 2014). In line with these results, Douglass et al. (1995) found high CART RNA expression in the MM of rats, but a later study failed to detect any CART RNA within the MB (Hurd and Fagergren 2000).

The human MB also showed CART RNA absence, in contrast to the anterior thalamus, where a high expression level of CART mRNA has been detected (Charnay et al. 1999; Hurd and Fagergren 2000).

### Neurotensin

Neurotensin (NTS), is a 13-amino acid neuropeptide, which plays a role in a plethora of functions and physiological processes: pain, thermoregulation, feeding, analgesia, blood pressure, arousal, sleep, and motivation. NTS has three known receptors: NtsR1, NtsR2, and NtsR3 (for review see Boules et al. 2013).

There is a lack of NTS in perikarya, but it has been found in a small number of fibers in the MM, with a much higher density in the caudal part of the rat MB (Kahn et al. 1982). It has been shown that the dorsal subiculum is the source of these projections and that the density of NTS-containing fibers in the MB is much higher in young rats, up to the third postnatal week (Kiyama et al. 1986). Moreover, a similar NTS pathway has been observed in infant humans: a high density of NTS-positive neurons in the subiculum, axons in the postcommissural fornix, and a large number of NTS-containing fibers in the MM (Sakamoto et al. 1986, 1987). The density of the NTS-positive structures is decreased markedly in the adult human MB (Langevin and Emson 1982; Mai et al. 1987), and only a small number of fibers containing NTS have been detected in the adult fornix (Roberts et al. 1983). The transient abundance of NTS in the MB of both species may suggest an important role of this peptide in the development of the hippocampal-MB circuit. Regarding NTS binding sites, the ML has been much richer in receptor presence in both, the rat and human (Boudin et al. 1996; Najimi et al. 2014).

### Neuropeptide Y

Neuropeptide Y (NPY), a 36-amino acid peptide, is connected to several CNS regulatory functions and processes, such as feeding behavior, modulation of synaptic transmission in learning and memory, neuroprotection, and regulation of proliferation of stem cells. The NPY expression is particularly increased in cell bodies of the hippocampus, amygdala, hypothalamus, periaqueductal gray, locus coeruleus, nucleus accumbens, basal ganglia as well as the cerebral cortex. In mammals, several types of NPY receptor has been identified (NPY1R- NPY6R). (For review, see Kautz et al. 2017; Shende and Desai 2020).

No cell bodies containing NPY and only its trace amounts in fibers located mostly in the ML characterizes the rat MB (de Quidt and Emson 1986; Ni et al. 2015). A similar distribution of NPY-positive structure has been observed in the anterior thalamus, while in the TNG, a moderate density of fibers and cell bodies containing NPY was detected, especially in the TNGd (Ni et al. 2015). Interestingly, however, studies on hamsters have shown that NPY is present in numerous neurons of both, the MM and ML, but only during pre- and perinatal periods of the development–they disappeared completely about the fifth day after birth. Dense fiber projections containing NPY were also visible in the main efferent routes of the MB, i.e., the mammillothalamic and mammillotegmental tracts, during these periods. The authors suggested that NPY may mediate axonal extension in the development of the CNS, and contribute to the establishment of connectivity between the MB and its main targets (Botchkina and Morin 1995). In regard to NPY receptors, NPY1R type has been detected in a dense network of processes and cell bodies in the median part of the MM, and in scattered processes in the MMl and ML. The most NPY1R-positive part of the ATN is the AM, where diffuse staining was found (Kopp et al. 2002; Wolak et al. 2003)—this pattern of NPY1R distribution matches the topographical organization of connectivity within the MB–ATN axis. The rat MB showed a low to moderate level of mRNA expression of various NPY receptors, NPY1R, NPY2R, NPY4R, and NPY5R; among the ATN, the highest expression level was characteristic for the AD (Parker and Herzog 1999).

In the human brain, no NPY or its mRNA has been found in the MB (Dudas et al. 2000; Dudas and Merchenthaler 2006; Escobar et al. 2004).

### Somatostatin

The somatostatin (SST) peptide family consists of two physiologically active forms of this neuropeptide: somatostatin-14 and somatostatin-28, both derived from pro-SST. SST function is associated with the inhibition of the growth hormone secretion in the hypothalamus, regulation of food intake, water intake, body temperature regulation, modulation of glucose content in blood, and motor activity. The SST-producing neurons in the CNS are primarily located in the hypothalamus (for review see Stegnel and Taché 2019). There are five main types of G protein-coupled SST receptors: sst1-sst5 (Schulz et al. 2000).

The MB of rats is virtually devoid of somatostatin—only a few fibers have been found in the structure, and a very similar pattern is characteristic for the ATN. In the TNG, some SST-positive cell bodies have been observed in the dorsal nucleus (Johansson et al. 1984; Lantos et al. 1995). In general, receptors for SST are present only in a neuropil of the rat MB, with moderate to high intensity of staining for sst1 and sst3; no sst2 has been detected in the MB (Dournaud et al. 1996; Hervieu and Emson 1998, 1999; Schindler et al. 1997; Uhl et al. 1985).

Similarly, the human MB lacks somatostatin (Bennett-Clarke and Joseph 1986; Filby and Gross 1983), but a dense group of SST-positive fibers has been observed in the ML in the infant brain (Najimi et al. 1989). Other studies have shown that the ML also contains SST mRNA (Mengod et al. 1992) and a moderate density of the SST-binding sites (Najimi et al. 1991). Only a very low density of SST receptors has been found in the human MB (Reubi et al. 1986).

### Orexins

Orexins (ORXs), sometimes referred to as hypocretins, are neuropeptides primarily linked to the regulation of food intake behavior, the wake-sleep cycle, and the arousal system in general. ORXs are synthesized in the lateral hypothalamus and in the junction of the hypothalamus and thalamus. Two types of ORXs were discovered—orexin A (ORXA) and orexin B (ORXB). Orexins bind to G protein-coupled receptors, orexin receptor type 1 (OX1R) and type 2 (OX2R). ORXA binds with high affinity to both orexin receptors, whereas ORXB selectively binds to OX2R (for review see Xu et al. 2013).

Although perikarya in the rat MB do not contain ORXs, both ORXA and ORXB have been observed in fibers, with exception of the MMl (Cutler et al. 1999; Nixon and Smale 2007). The ML exhibits a somewhat higher density of orexin-positive fibers, especially taking into account a comparison of orexin distribution among various species of rodents (Nixon and Smale 2007). In line with these results, also the presence of orexins’ receptors seems to be more pronounced in the ML, however, the results are ambiguous. Generally, mRNA for OX1R has not been found in the MB (Lu et al. 2000; Marcus et al. 2001), except for one study in which it was detected in a low density in the ML (Trivedi et al. 1998). On the contrary, a moderate to high density of OX2R mRNA has been found in both nuclei, except for the study of Trivedi et al. (1998), where no hybridization has been observed. Immunohistochemical observations are also vague. According to Suzuki et al. (2002), a moderate density of OX1R is present only in the ML, whereas in the study of Hervieu et al. (2001)—exclusively in the MM. Regarding OX2R, its presence has been shown in the whole MB, without any further distinction (Cluderay et al. 2002).

Studies concerning the anterior thalamus have brought equivocal results as well. A sparse density of both ORXs has been detected in fibers of the AD and AV (Nixon and Smale 2007) or the AV only (Cutler et al. 1999). Interestingly, mRNA of both orexin receptors, OX1R and OX2R, has been found exclusively in the AM (Marcus et al. 2001), while immunohistochemical detection conducted by Hervieu et al. (2001) showed that OX1R is present in the AD and AV in a density ranging from sparse to extensive. Likely, these varied results are the effect of different methods of detection used in the studies; nevertheless, it seems that there is a need to clarify the issue of the distribution of ORXs and their receptors in the MB–ATN axis.

### Galanin

Galanin is an amino acid peptide that exerts modulatory function on several physiological processes, such as glucose metabolism, feeding, nociception, learning, and memory. In the CNS, the biggest concentration of the galanin-positive cell bodies is contained within the hypothalamus, median eminence, locus coeruleus, and medial septal nucleus. In general, three groups of receptors for galanin have been identified: GalR1-GalR3 (for review see Robinson et al. 2006).

Except for a few single fibers, the rat MB virtually lacks galanin (Lantos et al. 1995; Skofitsch and Jacobowitz 1985; Takatsu et al. 2001) and the same is also true for humans (Dudas and Merchenthaler 2006; Gentleman et al. 1989). Concerning galanin receptors, GalR2 mRNA has been found at a high level in the rat MB, especially in the MM; whereas GalR1 mRNA is present only at a low level (Burazin et al. 2000; Mitchell et al. 1997; 1999).

## Other substances

In this section, we will describe two families of substances related to calcium ions, i.e., calcium-binding proteins and calcium sensor proteins.

### Calcium-binding proteins

Calcium-binding proteins (CaBPs) are a family of over 200 proteins specialized in the regulation of intracellular calcium concentrations. The mammalian brain is especially rich in three CaBPs: calretinin (CR), calbindin D28k (CB), and parvalbumin (PV) (e.g., Celio 1990). CR and CB are closely homologous proteins (Rogers 1987), which show very similar functions, such as neuroprotection against excitotoxicity and involvement in neuronal plasticity (e.g., D’Orlando et al. 2002; Jouvenceau et al. 1999; Schurmans et al. 1997; Schwaller 2014; Yuan et al. 2012). The neuroprotective role of PV is much less clear (D’Orlando et al. 2002; Waldvogel et al. 1991), and it is thought to take a part in regulating the local inhibitory effects of GABAergic interneurons (Schwaller 2009).

The distribution of CR and CB is somewhat complementary and mostly non-overlapping in the MB of the rat. CR is an excellent marker of the ML as it is present in almost all neurons of this nucleus, while in the MM, it is virtually absent (Jacobowitz and Winsky 1991; Resibois and Rogers 1992; Rogers and Resibois 1992). On the other hand, the majority of nerve cells in the MM are CB-positive (especially in the MMl), whereas those in the ML are practically devoid of CB (Celio 1990; Rogers and Resibois 1992; Sequier et al. 1990). Molecular studies on mice conducted by Mickelson et al. (2020) corroborate partially with these histochemical findings as the gene encoding CB has been found only in a neuronal population of the MMl. Moreover, CR-positive fibers in the MB participate in the formation of the mtt in rats, being the likely source of this particular CaBP in the anterior thalamus (Jacobowitz and Winsky 1991; Resibois and Rogers 1992). Indeed, thick bundles of CR-positive axons crossing the AM, together with neuropil containing CR in the AD have been detected (Arai et al. 1994; Résibois and Rogers 1992; Winsky et al. 1992). There is no evidence of CB presence in the mtt of rats, however strongly-immunostained neuropil for CB has been detected in the ventral part of the AV (e.g., Arai et al. 1994; Battaglia et al. 1992; Celio 1990; Rogers and Résibois 1992). The consensus has been established that these fibers and punctate structures in the AV continue with CB-positive axons in the mtt. It has been confirmed by Żakowski et al. (2014), by tracking the CB distribution in the MB–ATN axis of the guinea pig: from cell bodies in the MM, through axon fibers in the mtt, to terminal boutons in the ventral part of the AV. PV is also detectable in nerve cells of the rat MB: mainly in the MMm, and in lower numbers in the ML (Celio 1990). No PV has been observed in the mtt. However, strong PV-positive neuropil in the AD is the characteristic feature of the anterior thalamus of both, rats and guinea pigs (Arai et al. 1994; Celio 1990; Żakowski et al. 2013). In mice, neurons in the ML and MMm have been shown to contain PV-encoding gene, which is absent in the MMl (Mickelsen et al. 2020).

The presence of CaBPs in the neuropil of the MB, i.e., dense neuropillar immunoreactivity for CB and PV in the MM, and CR in the ML (Celio 1990; Jacobowitz and Winsky 1991; Resibois and Rogers 1992; Rogers and Resibois 1992) suggests an extrinsic source of these proteins. Dillingham et al. (2015) have studied two different MB inputs in this regard, from the TNG and hippocampal formation. It has appeared that in the case of PV and CB, the TNGv may be the main source of the neuropillar staining in the MM, as it contained a substantial number of PV- and CB-positive cells that send their axons to the MM. Meanwhile, CR was virtually absent in neurons projecting from the TNG to the MB. In contrast, CR has been found in a considerable number of cells in the postsubiculum, which efferents reach the ML. Otherwise, no CaBPs were observed in neurons projecting from the hippocampal formation to the MB of the rat. Thus, the authors suggest that PV and CB in projecting neurons of the TNGv may contribute to the functions of the MM, such as memory through theta rhythmical firing, whereas CR from the postsubiculum may play a role in modulation of the head-direction signal in the ML (Dillingham et al. 2015).

Numerous body cells containing CaBPs have also been observed in the MB of primates, especially in regard to PV and CR (Bernstein et al. 2007; Dixon et al. 2004; Fortin and Parent 1997; Xiao and Barbas 2002). Interestingly, while many CB-positive neurons have been found in the MB of rhesus monkeys and squirrel monkeys (Fortin and Parent 1997; Xiao and Barbas 2002), this protein is virtually absent in the MB of humans (Dixon et al. 2004; Sanghera et al. 1995).

### Calcium sensor proteins

Similar to calcium-binding proteins, neuronal calcium sensor proteins (NCSPs) are members of the EF-hand proteins superfamily. Their main function is to initiate and mediate intracellular signal cascades. Among many others, intracellular calcium-sensing proteins include visinin-like protein (NVP-1), NVP-2 and NVP-3, recoverin (visinin), neuronal calcium sensor-1 (frequenin), and hippocalcin (for review see Braunewell and Gundelfinger 1999).

By mapping gene expression in the rat brain, Paterlini and colleagues (2000) have revealed that the MB is one of the richest structures of NCSPs mRNA within the hypothalamus, especially in regard to hippocalcin and NVP-3, which a high mRNA level has been found mostly in the MM. Neuronal calcium sensor-1, NVP-1 and NVP-2 mRNAs were also present in the MB, but the expression was moderate (Paterlini et al. 2000). Initial studies concerning the distribution of visinin-like proteins in the rat brain, have shown a very characteristic pattern in the MB–ATN axis, i.e., numerous labeled cells in the MMl, fibers in the mtt, and a high density of fibers and terminal-like boutons containing NVP in the ventral part of the AV (Takami et al. 1985). The studied protein was referred to by the authors as visinin, so it is difficult to say which NVPs distribution was investigated. As visinin localization is restricted to the retina, pineal gland, and olfactory neurons (Braunewell and Gundelfinger 1999), and the immunoreactivity of NVP-2 is weak in the MB (Saitoh et al. 1994), the studied protein was most likely NVP-3 (or NVP-1). Nevertheless, NCSPs seem to have an important role in the rat MB–ATN axis, and probably in the whole extended hippocampal system, as numerous NVP-positive neurons have been found in the hippocampal formation and TNG as well (Kiyama et al. 1985; Takami et al. 1985). It has been shown that NCSPs may play various roles in the CNS, including involvement in memory and learning processes (for review see Groblewska et al. 2015). Study on human brains has failed to detect NVPs in the MB (Bernstein et al. 1999).

## Conclusions

For the past 40 years, many bioactive substances and their receptors have been found in the MB of the rat, including classical neurotransmitters, various neuropeptides and other substances, such as calcium-binding proteins. Undoubtedly, there is a need for continuation of such studies, as novel substances are still being discovered. Moreover, the modern neurochemical methods and much more specific antibodies give an opportunity to verify the results of the studies concerning chemical parcellation of the MB of rats, but also other species, including humans. Full knowledge of the MB neurochemistry may facilitate a better understanding of the structure functions and its complex relations with other structures of the extended hippocampal system.

## Data Availability

Not applicable.
